# Job Crafting and Work Engagement: The Mediating Role of Work Meaning

**DOI:** 10.3390/ijerph18105383

**Published:** 2021-05-18

**Authors:** Onintze Letona-Ibañez, Silvia Martinez-Rodriguez, Nuria Ortiz-Marques, Maria Carrasco, Alejandro Amillano

**Affiliations:** 1 Department of Social Psychology and Development, Faculty of Psychology and Education, University of Deusto, 48007 Bilbao, Spain; nortiz@deusto.es (N.O.-M.); alejandro.amillano@deusto.es (A.A.); 2 Department of Social Pedagogy and Diversity, Faculty of Psychology and Education, University of Deusto, 48007 Bilbao, Spain; silvia.martinez@deusto.es; 3 Department of Personality, Assessment and Psychological Treatment, Faculty of Psychology and Education, University of Deusto, 48007 Bilbao, Spain; mcarrasc@deusto.es

**Keywords:** positive organizational psychology, engagement, job crafting, work meaning, cognitive crafting, mediation

## Abstract

One of the most widely researched personal resources is job crafting, for which several studies have confirmed the existence of a positive relationship with engagement. Some authors suggest that it would be necessary to go deeper into the mechanisms that can help us explain this relationship. Therefore, the aim of this study is to ascertain the possible influence of the meaning of work on the relationship between job crafting and engagement. The sample is composed of 814 workers (50.4% women) with an average age of 41.68 years (SD = 9.78). The results were obtained by simple mediation analysis using PROCESS. The meaning of work mediates the relationship between job crafting and engagement, this influence being especially significant in the case of cognitive crafting. This study confirms the positive relationship between job crafting and engagement. However, in the case of some types of job crafting, increased levels of engagement only occur if the individuals also manage to increase the levels of meaning attributed to the work role. Therefore, in order to improve the well-being levels of working people, it would also be necessary to help them understand how these changes help them to attribute more meaning to their work.

## 1. Introduction

Work plays a major role in most people’s lives, constituting one of the aspects that contribute to living a full and healthy life. The experiences and learning we gain from work can potentially impact our well-being while also boosting human development, improving our perception of self-efficacy, worth, social support, competence or self-confidence [1]. Therefore, finding and supporting ways in which people could positively enhance their work experience can also help improve well-being [2].

The job demands–resources theory proposed by Bakker and Demerouti [3] established a theoretical framework for understanding this employee well-being. It suggests that there is a meaningful relationship between increasing job-related resources and higher levels of engagement. Within this model, job crafting has been one of the most widely studied personal resources and has become important in the study of employee well-being as it is significantly related to other central variables in the work context, like commitment and job satisfaction in the organizational context [4]. In this sense, many studies have corroborated a positive relationship between job crafting and engagement [5]. However, some authors highlight the need for further knowledge of the mechanisms underlying this relationship, such as the meaning attributed to work. 

In this regard, job crafting has been theorized as a process that can influence the meaningfulness of work [6]. When jobholders alter their tasks, work relationships or interactions, they aim to create meaningful experiences and as a result, modify their work experiences and how they understand them [1,7]. Likewise, these changes help them to define their identity in the work context, intending to align it with their personal identity [6,8]. In addition, being involved in personally meaningful work has been related to job engagement [9]. Thus, employees who value their work positively and find sources of personal meaning through it are more interested and involved in their field of activity [10]. Therefore, the meaning of work could be identified as one of the possible explanatory mechanisms in the relationship between job crafting and engagement.

Given the importance of continuing research on how the meaning of work can contribute to more engaged employees and how organizations can foster meaning at work through employee characteristics [11], the main objective of this study is to increase knowledge of the relationship between job crafting and engagement, in order to test the possible effect of the meaning that people give their job and work role on this relationship.

### 1.1. Job Crafting and its Conceptualizations

Job crafting is a concept which, although it has subsequently been used and adapted by various authors, was initially proposed by Wrzesniewski and Dutton [12]. These authors sought to break with the conceptualizations that had been made of work contexts and proposed that—even in the most routine jobs and within the most restrictive environments—workers have the possibility of changing some aspects of their work. They define the concept of job crafting as an active behavior whereby workers try to change—through physical and/or cognitive modifications—the tasks or personal relationships within their work environment. Wrzesniewski and Dutton [12] identify three forms of job crafting: task crafting, related to the work tasks or activities that the person could modify in terms of quantity, type or scope of performance; cognitive crafting, which implies a change at the cognitive level in the way the person understands their job—whether as a grouping of independent tasks or as a set of integrated tasks with a unified meaning; and relational crafting, which refers to the social relationships that occur at work, and can modify the quantity and/or quality of these interactions.

Tims and Bakker [13] took up the original definition of job crafting made by Wrzesniewski and Dutton and intended to complement this model, because they considered it too generalist, thus proposing the currently predominant theoretical model of job crafting. These authors integrated job crafting into the demands and resources model and proposed four categories: increasing structural job resources, decreasing hindering job demands, increasing social job resources and increasing challenging job demands.

Job crafting is, according to both models, a process of autonomous and proactive change that the worker carries out when they understand that the realization of these changes is possible. However, each of these models proposes different dimensions as a basis for understanding job crafting.

For this reason, this study proposes to evaluate job crafting with the aim of obtaining more information from both theoretical approaches and the proposed dimensions.

### 1.2. Job Crafting and Engagement

The theoretical model of engagement on which this study is based was developed by Schaufeli and Bakker [14]. According to these authors, engagement is a persistent cognitive-affective state over time characterized by three dimensions: vigor, dedication and absorption [15]. Based on this conceptualization, Bakker and Demerouti [16] developed the Model of Work Engagement that focused on the motivational process and on the role that personal and job resources play as precursors that facilitate experiencing high levels of engagement.

In this context, the relationship between job crafting and engagement has been one of the most widely studied and the positive relationship between these two variables has been shown in different studies [17,18,19,20]. This relationship has been confirmed in studies with working teams [21], studies conducted in different cultural contexts [22,23] and in several longitudinal studies like those by Nielsen and Abilgaard [24] and Tims et al. [25]. They showed that increasing job and social resources, together with the search for job challenges, predicts higher engagement.

In recent years, different review articles have focused on the variables related to job crafting, one of the broadest of which was the meta-analysis authored by Rudolph et al. [5]. This meta-analysis revised 122 independent samples and concluded that job crafting is positively related to engagement, among other variables related to the work context.

As a starting hypothesis for this study—in which we aim gain deeper insight into the relationship between job crafting and engagement—we hypothesize that:

**Hypothesis** **1** **(H1).** *Job crafting is positively related to engagement.*

### 1.3. Job Crafting and Work Meaning

Work meaning in this study is based on the theoretical framework of Steger and Dik [26], in which two factors stand out: on the one hand, the person’s understanding of their work environment and—once their identity, skills and needs are understood—the adjustment of their own person to this environment. On the other hand, framing work in the pursuit of a specific purpose also helps the person to better understand their work environment (tasks, purpose, work relationships, etc.), to strengthen their personal identity and self-knowledge and, therefore, to better understand their fit within the organization. Through work meaning, the person gets to know themselves better and brings meaning to their life.

In spite of being one of the variables that the original authors of job crafting, Wrzeniewski and Dutton [12], proposed as central to its understanding, and which other authors later recognized as fundamental to employees’ well-being at work [27], few studies have connected job crafting with the meaningfulness of one’s job.

Tims et al. [28] conducted one of these studies which was carried out with 114 employees who were evaluated for three consecutive weeks. The authors found that increasing job crafting was related to higher levels of job meaning and was always directed to optimizing employees’ perception of the fit between their job demands and available resources. Similarly, a recent study by Vermooten et al. [29] with 391 finance employees in South Africa showed that job crafting and work meaning are related and also underscored the predictive value of job crafting on the meaning variable.

The changes made by workers in their roles and functions may facilitate an increase in the meaning they attribute to their work, and therefore, this study proposes the following hypothesis:

**Hypothesis** **2** **(H2).** *Job crafting is positively related to work meaning.*

### 1.4. Engagement and Work Meaning

Although there is little empirical evidence, several studies have examined the relationship between work meaning and engagement.

May et al. [9] conducted one of the first empirical studies that suggested the existence of this relationship. These authors tested Kahn’s [30] theoretical approach in a study with 213 workers. Of the three variables established as prerequisites for engagement proposed by Kahn (availability, safety, meaning), they found that work meaning showed the strongest relationship. Further studies have helped to confirm that work meaning is a significant predictor of engagement [31,32].

At the moment, there are not many studies using large samples. In a study with 625 services employees, a positive and significant relationship was found between meaningfulness at work and engagement, suggesting a model in which work meaning acted as a predictor of engagement. This relationship was observed to be stronger in participants with high levels of well-being [33]. A second study with 443 participants also confirmed the strong and positive relationship between these two variables [34]. Likewise, in Fairli’s [35] study with a sample of 574 employees, meaningfulness at work was shown to have the strongest relationship with engagement, above other personal and organizational variables such as organizational support, peer and/or supervisor relationships or intrinsic rewards (like autonomy, task identity, self-efficacy, etc.)

Since available evidence shows that work meaning seems to positively influence the levels of engagement shown by the employee and as one of the steps in the process of gaining a better understanding of the relationship between these variables, we hypothesize that:

**Hypothesis** **3** **(H3).** *Work meaning is positively related to engagement.*

### 1.5. Work Meaning and Its Mediating Role

Some studies have highlighted the mediating role that work meaning can play in the relationship between different job characteristics or resources and engagement [31,36].

More specifically, some studies have found that work meaning can function as a mediator between variables conceptually close to job crafting. Perhaps the first mention of this type is to be found in Hackman and Oldham’s [37] job characteristics theory, which is one of the reference frameworks for job design. These authors proposed that for an employee to experience high intrinsic motivation, job satisfaction, job performance and low absenteeism and turnover intention, three states had to be fulfilled: firstly, employees must perceive their work as meaningful, secondly, they must feel responsible for the outcomes of their work and, thirdly, they must have knowledge of its results. Therefore, these authors had already suggested that work meaning is key to achieving employee well-being. Furthermore, they added that three job characteristics such as skill variety (the various skills and talents workers are required to develop), task identity (the degree to which jobholders identify and complete a workpiece with a visible outcome), task significance (the degree to which the job affects other people’s lives) are prerequisites for the perception of one’s work as meaningful. The proposal set out in this theory was substantiated years later through a meta-analysis in which 259 studies were analyzed. It affirmed that work meaning was the strongest mediator between some of the job characteristics analyzed. These included skill variety, task understanding and its impact, and positive work outcomes such as performance, job satisfaction and intrinsic motivation [38].

Later studies yielded similar results, demonstrating that work meaning had a mediating influence on predictive variables such as peer relationships, job characteristics, feedback, skills development and utilization or the work-role fit and engagement [9,36,39,40]. Asiwe et al. [34] showed that the positive relationship between engagement and job design—as a job characteristic or resource—was mediated by the perceived meaning. More recently, Bakker and Albrecht [41] proposed job crafting as an appropriate strategy to increase engagement as it also enhances the meaningfulness experienced by the worker and the fit between job demands and the person’s resources to address the task.

Continuing in the line of the most recent studies and proposals, it is considered that work meaning can be a valid mediator that helps to gain better understanding of the relationship between job crafting and engagement. For this reason, the following is proposed:

**Hypothesis** **4** **(H4).** *Work meaning mediates the relationship between job crafting and engagement.*

## 2. Materials and Methods

### 2.1. Participants

The sample consists of 814 employees (50.4% women) with an average age of 41.68 (SD = 9.78) and ages ranging between 22 and 71 years. Of the subjects, 63.4% are between 35 and 54 years of age whereas 10.9% could be considered older workers, i.e., over 55 years of age.

Most of the sample (86.5%) are university graduates and very few participants report having completed only secondary or lower educational qualifications (3.5%).

As regards their job category, 79.8% occupy positions that could be considered for highly qualified employees (executive and management or technical jobs). Furthermore, 81.1% are employed workers, mainly in private organizations (78.7%) and are full-time employees with permanent contracts (87.2%), often exceeding 35 work hours/week (86%). Over half of the participants report gross incomes of more than 24,000 euros per year (66.3%). A large number of participants report that they have been working for more than 10 years (73.3%), and 38.5% of this group have been working more than 20 years.

### 2.2. Procedure

The sample was collected by using two different methods: snowball sampling was used to access some subjects, and other possible participants were contacted through a job-related and active job search media site to reach employees in a random manner. In both cases, participants signed an informed consent before accessing the questionnaire. It is important to note that this sampling system, as well as all the procedures used in this study, have received the approval of the University of Deusto Ethics Committee (Ref. ETK-23/17-18).

Once all the responses had been collected and the invalid or duplicate entries had been removed, the data were then included in a database for analysis.

### 2.3. Instruments

Job Crafting (Job Crafting Questionnaire, JCQ). Based on the theoretical background presented by Wrzesniewski and Dutton [12], job crafting has been measured using the Spanish version of the Job Crafting Questionnaire [42] by Slemp and Vella-Brodick [43] which consists of 15 items that measure three dimensions: task crafting, relational crafting and cognitive crafting, using a 6-point Likert scale (1 = almost never, 6 = very often) with a reliability index of 0.86 for this study.

Job Crafting (Job Crafting Scale, JCS). Based on the theoretical background presented by Tims and Bakker [13], assessment was performed with the Job Crafting Scale [44] comprised of 4 dimensions (increasing structural job resources, decreasing hindering job demands, increasing social job resources and increasing challenging job demands) measured through 21 items, with a 5-point Likert scale (1 = never, 5 = often). Cronbach’s alpha for this study is 0.77.

Work Engagement (Utrecht Work Engagement Scale-9, UWES-9). The Spanish version of the UWES-9 [45], which was translated by these same authors, has been used. It assesses work engagement through three constituting aspects, vigor, dedication and absorption, consisting of three items each, and measures using a 7-point Likert scale (1 = never, 7 = always), with Cronbach’s alpha of 0.90.

Work Meaning (Work and Meaning Inventory, WAMI). It has been evaluated by using the Spanish translation of the WAMI by Steger, Dik Duffy [46]. This tool consists of 10 items and it is based on a 5-point Likert scale (1 = totally untrue, 5 = totally true) with a reliability index of 0.84.

### 2.4. Data Analysis

SPSS (version 26.0 for Windows; IBM Corp., Armonk, NY, USA) was used for the descriptive analysis of the variables in this study. Additionally, different simple mediation analyses were run to test the effect of work meaning in the relationship between job crafting and engagement. The macro PROCESS (version 3.3) and Hayes’s procedure [47] were used for this purpose. PROCESS model 4 has been used specifically for simple mediations. As proposed by Hayes [47], the analysis was repeated for each of the independent variables (dimensions), including in each case the rest of the dimensions of the model and control variables (age, education level, job position, income, years of work and seniority in current position) as covariates. The direct and indirect effects between the different variables were examined in all cases with the bootstrapping method (confidence interval of 95% and using 10,000 samples from different bootstraps). Some of the socio-demographic variables were included as control variables due to their significant relationship with engagement.

Following Baron and Kenny’s [48] initial proposal, mediation is checked to determine whether it is complete or partial. However, as Hayes [47] pointed out, this affirmation would not be sufficient, so reference is also made to the size of the indirect effect found. Many statistical indicators have been proposed in recent years as appropriate for informing on the size of the mediation effect, although there is still no clear consensus [47,49]. In this study, the completely standardized value of the indirect effect is referred to, which allows us to compare the weight of the indirect effect of each of the predictor variables in each model [47]. Moreover, the proportion mediated is included, which indicates what percentage of the total effect is due to mediation. It is one of the most widely used measures of effect size in samples with over 500 subjects [50,51].

## 3. Results

With regard to the relationship between job crafting and engagement, this study confirms the existence of a positive and significant relationship between the two variables, taking into account the two explanatory models of job crafting (see Table 1). 

All the dimensions and global score of the JCQ evidence a positive and significant relationship with the total score for engagement and each of its three dimensions. The highest correlations are found in the relationship between engagement and the JCQ global score, with correlation coefficients between 0.55 and 0.45. The JCQ dimension that shows the highest correlations with engagement is cognitive crafting (*r* = 0.50), followed by task crafting (*r* = 0.45) and relational crafting (*r* = 0.33). Cognitive crafting is also the dimension which shows the strongest relationship with each of the dimensions of engagement: vigor (*r* = 0.46), dedication (*r* = 0.48) and absorption (*r* = 0.42).

When analyzing the JCS global score and dimensions, it is noted that the dimension of increasing job challenge demands has a higher correlation with engagement (*r* = 0.48) and also with each of its dimensions: vigor (*r* = 0.44), dedication (*r* = 0.45) and absorption (*r* = 0.40). The rest of the JCS dimensions and global score also show significant and positive correlations with engagement, except for the dimension of decreasing job demands, which evidenced no significant relationships.

Both job crafting models also showed positive and significant relationships with work meaning. Correlations between 0.38 and 0.63 were observed in the case of the JCQ, highlighting the correlation between work meaning and the dimension of cognitive crafting, which is 0.63. Work meaning also evidences positive and significant relationships in the JCQ global score and dimensions although with much lower correlations (between 0.38 and 0.16). The dimension of increasing job challenge demands presents a stronger relationship with work meaning (0.38). The dimension of decreasing job demands of the JCS is also an exception in this case since it does not show a significant relationship with work meaning.

When focusing on the relationship between engagement and work meaning, significant and positive correlations can be observed. The relationship between the global score for engagement and work meaning (0.73) is remarkable, with the dimension of dedication showing a higher correlation (0.75).

### The Mediating Effect of Work Meaning in the Relationship between Job Crafting and Engagement

Simple mediation analysis was performed, analyzing the two theoretical job crafting models separately, in order to fulfil the main aim of this study and test the mediating influence of work meaning in the positive and well-established relationship between job crafting and engagement. The first included the three dimensions of job crafting proposed in Wrzeniewski and Dutton’s [12] model as precedent or predictor variables and the second included the four dimensions of job crafting proposed in Tims and Bakker’s [13] theoretical model as predictors of job crafting.

**Figure 1:**
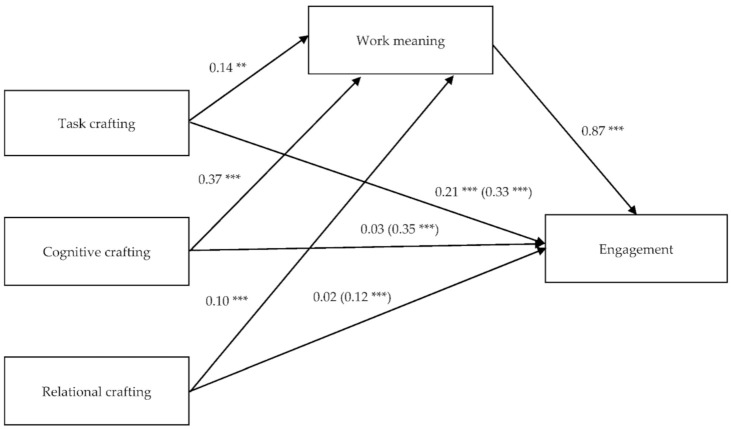
Simple mediation model between the dimensions of the JCQ and engagement, with work meaning as mediating variable. Non-standardized values. ** *p* < 0.01, *** *p* < 0.001.

The first simple mediation model that was analyzed, which includes the three dimensions of the JCQ as predictor variables, is shown in Figure 1.

In the model, we observe that the predictor variables show a significant relationship with the mediator variable: task crafting (b = 0.14, SE = 0.03, t(804) = 4.42, *p* < 0.001), cognitive crafting (b = 0.37, SE = 0.02, t(804) = 16.27, *p* < 0.001) and relational crafting (b = 0.10, SE = 0.02, t(804) = 4.43, *p* < 0.001). Likewise, the relationship between work meaning as mediator variable and engagement as result variable was also significant (b = 0.87, SE = 0.04, t(803) = 20.61, *p* < 0.001). Table 2 describes the direct and total effects of the dimensions of the JCQ on engagement and Table 3 shows the results for the indirect effects, taking into account the effect of the work meaning variable.

As can be seen in Table 3, the indirect effects are significant for the three dimensions of the JCQ. This indicates that work meaning has a mediating effect on the relationship between the three dimensions of the JCQ and engagement. Furthermore, for cognitive crafting and relational crafting, the direct effect with engagement is very close to 0 and is no longer significant when the mediating role of work meaning is controlled (b = 0.03, SE = 0.03, *p* = 0.313 for cognitive crafting and b = 0.02, SE = 0.03, *p* = 0.355 for relational crafting), we would be talking about partial mediation in the case of task crafting. Although the direct effect is still significant (b = 0.21, SE = 0.04, *p* < 0.001), it is lower than the corresponding total effect (b = 0.33, SE = 0.04, *p* < 0.001).

As regards the effect size, work meaning seems to have a greater effect on the relationship between cognitive crafting and engagement, with a larger completely standardized indirect effect over the other two dimensions (β = 0.32, SE = 0.02, 95% IC = 0.270, 0.370), accounting for 92% of the total effect (PM = 0.92).

**Figure 2:**
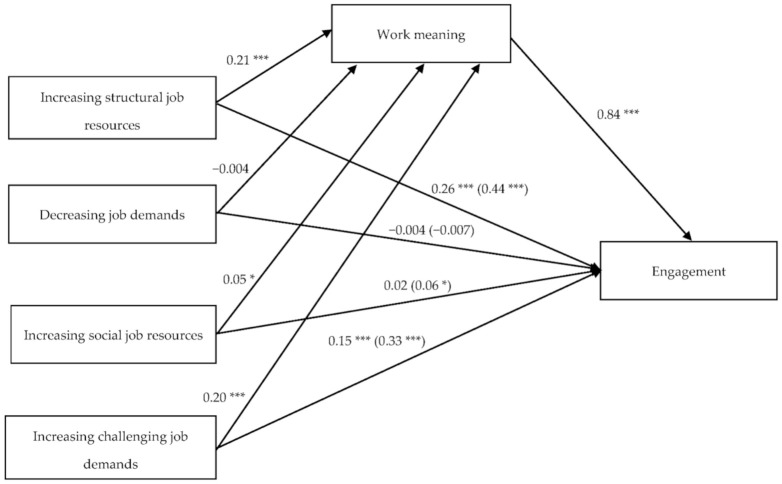
Simple mediation model between the dimensions of the JCS and engagement, with work meaning as mediating variable. Non-standardized values. * *p* < 0.05, *** *p* < 0.001.

In the second simple mediation model studied, the four dimensions of the JCS are shown as predictor variables of engagement while the mediating role of work meaning in this relationship is also tested (see Figure 2).

It can be observed how the dimensions of the JCS, except the dimension of decreasing job demands (b = −0.004, SE = 0.02, t(803) = −0.16, *p* = 0.870), show a significant relationship with the mediator variable: increasing structural job resources (b = 0.21, SE = 0.04, t(803) = 4.86, *p* < 0.001), increasing social job resources (b = 0.05, SE = 0.02, t(803) = 2.35, *p* < 0.05) and increasing challenging job demands (b = 0.20, SE = 0.03, t(803) = 6.60, *p* < 0.001). Work meaning, the mediator variable, also shows a significant relationship with engagement in this model (b = 0.84, SE = 0.03, t(802) = 25.05, *p* < 0.001).

The direct and total effects for each of the dimensions of the JCS in relation to engagement can be seen in Table 4, while Table 5 describes the indirect effects. As regards the latter, all the dimensions of the JCS except decreasing job demands (b = −0.003, SE = 0.02, 95% CI = −0.040, 0.033) show significant indirect effects, which means that work meaning plays a mediating role in each of these dimensions with engagement. If we examine each of the analyses, we see how this mediating effect can vary for each of the dimensions. Mediation is partial for the dimensions of increasing structural job resources and increasing job challenge demands, since the direct effect remains significant (b = 0.26, SE = 0.04, *p* < 0.001; b = 0.15, SE = 0.03, *p* < 0.001, correspondingly) although lower in comparison to the total effect. The data indicate total mediation for the dimension of increasing social job resources since the direct effect is no longer significant when the role of the mediator variable is controlled (b = 0.02, SE = 0.02, *p* = 0.390). However, the total effect of this dimension on engagement is very low (b = 0.06, SE = 0.06, *p* < 0.05), which also limits the explanatory effect of this mediation.

As regards the size of these effects, the relationship between increasing challenging demands and engagement is where work meaning seems to have greater influence (β = 0.16, SE = 0.02, 95% CI = 0.104, 0.209) accounting for 51% of the relationship (PM = 0.51).

## 4. Discussion

This study aims to explore the mechanisms that explain the relationship between job crafting and engagement and proposes that work meaning is one of the factors that may influence this relationship. As in other studies [5,17,18,24,25,52], our research shows the existence of a positive relationship between job crafting and engagement, leading us to confirm the first proposed hypothesis (Hypothesis 1).

Focusing on a more in-depth study of the relationship between each of the dimensions of job crafting and engagement, we find that the dimension of cognitive crafting—from Wrzesniewski and Dutton’s [12] theoretical model—shows a stronger relationship with this variable.

Other studies have confirmed the relationship between the cognitive crafting dimension and engagement-related variables such as organizational commitment [4], job satisfaction [4,43,53] or psychological and subjective well-being [53]. Fewer studies have proven the relationship between cognitive crafting and engagement. In this sense, the scarce literature supports the results found in our study. In this regard, Sakuraya et al. [54] proved the effectiveness of a job crafting intervention program on work engagement and concluded that the program promoted work engagement especially through cognitive crafting. Other authors have also emphasized the fundamental role of cognitive crafting as a predictor of work engagement [55,56]. The relevance of this relationship can be explained by taking into account that job crafting, more specifically its cognitive dimension, allows the person to carry out a process of reinterpretation of their work context through which aspects related to engagement (such as enthusiasm, inspiration or challenge) can be reinforced.

In our study, job crafting presents a clearly positive relationship with work meaning, thus confirming Hypothesis 2. Wrzesniewski and Dutton [12] believed from the start that the increase in work-related meaning could be one of the direct results of job crafting. They pointed out that when employees introduce job changes, whether they are related to tasks, peer relations or their view of job roles, they are pursuing meaningfulness and a better person–job fit. Other authors have later demonstrated the potential of job crafting as a tool to create meaning on a personal level that helps individuals to understand their most immediate work environment [1,6,57]. There are still few studies that can support our results [28,29].

Focusing on the analysis of the different dimensions of job crafting, we find a stronger relationship with work meaning in the JCQ dimensions. In addition, cognitive crafting is particularly outstanding among them since it shows a closer relationship with work meaning in comparison to the rest of the dimensions. These results support those presented by Geldenhuys et al. [58] in a study in which, on a weekly basis, cognitive crafting was shown to be a predictor of higher levels of meaningfulness. In a complementary manner, the dimensions of increasing challenging job demands and increasing structural job resources were found to be the JCS dimensions that showed a stronger relationship with work meaning. These results partially coincide with the findings of Petrou et al. [59], as in this case, only the dimension of increasing structural job resources presented a relationship with meaning-making at work. It is interesting to note the positive relationship that both cognitive crafting and the dimension of increasing challenging job demands, has shown with work meaning. For the former, the same process of changing perspective allows the person to identify with certain values or objectives that connect with their identity and to understand the value of their work role, both at the individual and community level. For the latter, it may seem that the fact of facing novel projects or tasks not only tests the person’s abilities and skills, but that this process seems to allow them to connect with aspects central to their identity, such as the perception of self-efficacy or competence. In the case of dimensions related to social aspects, although peer relations and their results such as stronger group membership or higher perceived social support have been identified as a major source or meaning [27], altering work-context-related social interactions does not show a strong relationship with greater work meaning in our study in spite of a significant relationship being found. It may be that in the case of social relationships at work, what is significant for work meaning is not the increase or limitation of the number of relationships, but the framework in which these are understood and how this affects the person. This aspect can be identified more like a process of cognitive crafting.

Regarding the relationship between work meaning and engagement, our results show a positive relationship between these two variables, thus confirming Hypothesis 3 of this study. Our results therefore ratify the evidence from previous studies that highlighted the relationship of work meaning with engagement as one of the strongest [9,31,33,34,35,60] and also support Kahn’s theoretical approach to engagement, in which perceiving work as meaningful is one of the three prerequisites for experiencing high levels of engagement [30]. Recently, Han et al. [61] have proposed that the positive relationship between work meaning and engagement responds to the proposal made by Fredrickson´s broaden-and-build theory. They explain that the person who perceives their work as meaningful, generates a framework of thought and action which in turn helps the person to reinforce their personal resources and thus increase engagement.

In addition to checking the relationship between the different variables, our research aim was to gain a deeper understanding of the relationship between two of the principal variables in the framework of positive organizational psychology: job crafting and engagement. As we have stated, this relationship has been widely studied and supported by different theories. However, in spite of the direct relationship between both variables having been confirmed, the mechanisms that influence it remain to be explained. They will allow us to understand why the changes employees make in their work environment, whether they are related to their tasks or resources, relationships or their perspective on their work, have a positive impact on their level of engagement.

In relation to this, our results have confirmed the existence of a mediating effect of work meaning in the relationship between job crafting and engagement, thus confirming Hypothesis 4 and postulating work meaning as one of the explanatory mechanisms in this relationship. Therefore, when workers decide to make changes in their jobs, whether they are related to tasks, resources, peer interaction or perception, this does not directly contribute to higher levels of engagement or commitment. However, these changes help employees to reset the perception they have of their job and to understand it from a point of view that is more meaningful for them. An understanding of one’s own job as meaningful is what helps these changes to contribute to higher levels of engagement.

Our findings can be contrasted with those of other studies, although to date not many have addressed this question. Dan et al. [11], with a sample of 1151 firefighters, found that job crafting could influence engagement levels through the meaning attributed to the job. Furthermore, Vermooten et al. [29] conducted a study with 391 participants and confirmed a model in which a proactive personality influences employees’ job crafting levels. This, in turn, impacts work meaning, which is expressed as higher levels of engagement and lower turnover intention. Although these proposals are close to the one in this study, the job crafting variable was measured using Tims and Bakker’s theoretical approach, which is specifically the one that does not recognize work meaning as a result of the modifications that workers make in their jobs.

In an attempt to add knowledge to that proposed by previous authors, this study analyzes the role of job crafting dimensions and our results indicate that the effect of work meaning on the relationship between job crafting and engagement is different according to the type of job crafting or modifications that people make in their job environments.

First of all, the mediation of work meaning could not be confirmed in the case of the JCS dimension of decreasing job demands. This dimension showed a different relationship pattern to the rest of the job crafting dimensions analyzed, evidencing no relationship with engagement and work meaning. While it may seem surprising, these results confirm those of previous studies such as Tims et al. [17], which created the JCS, or those by Bakker et al. [44] and Sora et al. [62] for adaptation of the JCS. In said studies, this category of job crafting systematically showed behavior that was totally opposite from the rest of the dimensions as regards the relationship with the other variables analyzed. It can be understood that trying to minimize or eliminate the demands of your job is not culturally accepted and therefore not a strategy used by workers to increase their work well-being.

Secondly, our results have shown that in the case of job crafting dimensions related to social relationships at work (relational crafting and increasing social job resources), although the relationship with engagement is more limited compared to other dimensions, the mediating effect of work meaning is significant, largely helping to explain this relationship. Social relationships are one of the main sources of meaning in the work context [27] and, therefore, aspects such as group membership, positive relationships, social support or contact with the people receiving their services can help workers to understand their work as meaningful and, therefore, experience higher levels of engagement.

Lastly, it is worth mentioning that the effect that work meaning has on the relationship between job crafting and engagement is particularly relevant in the case of cognitive crafting. When workers change the perception they have of their job or their work role, in other words, when they understand that each task or activity they perform is meaningful within the entire process and not just separate activities, they achieve an overall understanding of their work which enables them to give meaning to their work role and, as a result, they can feel more committed to their work. As Wrzesniewski and Dutton [12] have already pointed out, the cognitive component of job crafting is vital in jobholders’ processes of altering their work environment since it is precisely that shift in perspective that helps to make their work meaningful and strengthens their personal identity.

## 5. Conclusions

This study was designed with the objective of expanding the knowledge that we have today of the job crafting variable and of the relationship dynamics that it presents with other important variables within the framework of work well-being, such as engagement and work meaning. Evaluating the job crafting variable from two different theoretical frameworks has allowed us to observe their complementarity and deepen the knowledge of the behavior of each of the dimensions of this variable. The role of cognitive crafting stands out above the rest, being the dimension that has shown stronger relationships and a higher predictive value in relation to engagement and meaning at work.

However, the essential objective of this research, and therefore also its greatest contribution, has been the verification of the mediating role of the meaning attributed to work in the relationship between job crafting and engagement. Our results confirm that in order for the modifications that people make in their work and their work role—modifying the tasks, selecting their social relationships or putting their skills into practice—to lead to higher levels of engagement, it is important that people can perceive their work as meaningful. This influence seems to be more relevant precisely when people change the perception they have of their work, that is, when they make modifications at the cognitive level. This is also the case when the changes they make are related to the interactions and social relations that take place in the work environment.

The positive influence that job crafting can have on the work environment is becoming increasingly clear, bringing benefits at both the individual and organizational levels. Taking into account the results obtained in this research, we believe that these benefits could be greater if the individual were helped to make changes in the cognitive perception they have of their work. Organizations could make it easier for their workers to understand the purpose of the activities they carry out, and help them understand that all of the tasks they perform on a daily basis have a common meaning and are part of a single objective. In this way, organizations would help their employees experience higher levels of engagement and attribute meaning to their work, thus ensuring other benefits at the organizational level, such as higher levels of performance, less work stress and lower levels of absenteeism or intention to leave.

### Limitations and Future Lines of Research

First, we must refer to the nature of the sample. We find that a sample with certain socio-demographic characteristics that may limit its representativeness. This is because our sample is mainly composed of white-collar workers and the results of our study should be considered representative of this group of workers. Additionally, the transversal design of the study and the fact of having used self-administered scales are also limitations that must be taken into account when generalizing the results.

In the future, there is a need to continue accumulating evidence that allow us to confirm the results found in this investigation. On the one hand, it would be interesting to have access to samples of workers with different characteristics that would help complete and extend these results. On the other hand, it would be interesting if the adaptations with Spanish sample could be used in future studies and with samples of different socio-demographic characteristics. In this way, we would advance in confirming the factor structure of these scales and we would obtain more evidence of its validity.

## Figures and Tables

**Table 1:** Correlations between engagement, job crafting, work meaning and their corresponding dimensions and reliability indexes (Cronbach’s alpha) for each of the variables and their dimensions.

	Variables	1	2	3	4	5	6	7	8	9	10	11	12	13	
1.	Engagement	(0.917)													
2.	Vigor	0.91 ***	(0.829)												
3.	Dedication	0.93 ***	0.82 ***	(0.884)											
4.	Absorption	0.86 ***	0.63 ***	0.68 ***	(0.756)										
5.	Task Crafting (JCQ)	0.45 ***	0.41 ***	0.43 ***	0.37 ***	(0.754)									
6.	Cognitive Crafting (JCQ)	0.50 ***	0.46 ***	0.48 ***	0.42 ***	0.43 ***	(0.864)								
7.	Relational Crafting (JCQ)	0.33 ***	0.32 ***	0.31 ***	0.26 ***	0.35 ***	0.40 ***	(0.791)							
8.	JCQ Total	0.55 ***	0.52 ***	0.52 ***	0.45 ***	0.72 ***	0.82 ***	0.77 ***	(0.862)						
9.	Increasing structural job resources (JCS)	0.45 ***	0.41 ***	0.45 ***	0.35 ***	0.45 ***	0.30 ***	0.18 ***	0.39 ***	(0.796)					
10.	Decreasing hindering job demands (JCS)	−0.01	0.002	0.001	−0.03	0.002	0.07 *	−0.01	0.03	0.05	(0.778)				
11.	Increasing social job resources (JCS)	0.18 ***	0.14 ***	0.18 ***	0.17 ***	0.22 ***	0.23 ***	0.31 ***	0.33 ***	0.13 ***	0.11 **	(0.766)			
12.	Increasing challenging job demands (JCS)	0.48 ***	0.44 ***	0.45 ***	0.40 ***	0.61 ***	0.35 ***	0.37 ***	0.55 ***	0.51 ***	−0.03	0.28 ***	(0.744)		
13.	JCS Total	0.38 ***	0.34 ***	0.37 ***	0.37 ***	0.45 ***	0.36 ***	0.33 ***	0.48 ***	0.54 ***	0.56 ***	0.69 ***	0.63 ***	(0.773)	
14.	Work Meaning	0.73 ***	0.64 ***	0.75 ***	0.59 ***	0.42 ***	0.63 ***	0.38 ***	0.63 ***	0.33 ***	−0.005	0.16 ***	0.38 ***	0.30 ***	(0.899)

Note. * *p* < 0.05, ** *p* < 0.01, *** *p* < 0.001.

**Table 2:** Direct and total effects of the dimensions of the JCQ on engagement.

JCQ Dimensions	Direct Effect (c’)					Total Effect (c)				
	B	SE	*t*	LL	UL	B	SE	*t*	LL	UL
Task crafting	0.21	0.04	5.51 ***	0.136	0.287	0.33	0.04	7.11 ***	0.242	0.426
Cognitive crafting	0.03	0.03	1.01	−0.029	0.093	0.35	0.03	10.51 ***	0.287	0.419
Relational crafting	0.02	0.03	0.92	−0.030	0.082	0.12	0.03	3.36 ***	0.049	0.186

Note. Non-standardized values. Confidence interval of 95%. LL = lower limit of the interval, UL = upper limit of the interval. *** *p* < 0.001.

**Table 3:** Indirect effects of the dimensions of the JCQ on engagement.

JCQ Dimensions	B	SE	LL	UL	β	SE	LL	UL	PM
Task crafting	0.12	0.03	0.057	0.185	0.08	0.02	0.040	0.129	0.37
Cognitive crafting	0.09	0.02	0.040	0.143	0.32	0.02	0.270	0.370	0.92
Relational crafting	0.32	0.02	0.270	0.375	0.08	0.02	0.037	0.130	0.72

Note. B = non-standardized values, β = standardized values. Confidence interval of 95%. LL = lower limit of the interval, UL = upper limit of the interval. PM = proportion mediated.

**Table 4:** Direct effects and total effects of the dimensions of the JCS on engagement.

JCS Dimensions	Direct Effect (c′)					Total Effect (c)				
	B	SE	*t*	LL	UL	B	SE	*t*	LL	UL
Increasing structural job resources	0.26	0.04	6.17 ***	0.178	0.345	0.44	0.05	7.92 ***	0.332	0.551
Decreasing job demands	−0.004	0.02	−0.17	−0.048	0.40	−0.007	0.030	−0.234	−0.066	0.052
Increasing social job resources	0.02	0.02	0.86	−0.022	0.057	0.06	0.06	2.20 *	0.006	0.113
Challenge demands	0.15	0.03	5.12 ***	0.096	0.216	0.33	0.04	8.3 1 ***	0.251	0.407

Note. Non-standardized values. Confidence interval of 95%. LL = lower limit of the interval, UL = upper limit of the interval. * *p* < 0.05, *** *p* < 0.001.

**Table 5:** Indirect effects of the dimensions of the JCS on engagement.

JCS Dimensions	B	SE	LL	UL	β	SE	LL	UL	PM
Increasing structural job resources	0.18	0.04	0.101	0.260	0.11	0.02	0.061	0.160	0.40
Decreasing job demands	−0.003	0.02	−0.040	0.033	−0.003	0.02	−0.041	0.033	−0.01
Increasing social resources	0.04	0.02	0.007	0.079	0.05	0.02	0.009	0.092	0.70
Challenge demands	0.17	0.03	0.114	0.234	0.16	0.02	0.104	0.209	0.51

Note. B = non-standardized values, β = standardized values. Confidence interval of 95%. LL = lower limit of the interval, UL = upper limit of the interval. PM = proportion mediated.

## Data Availability

All files are available from the Figshare database (https://figshare.com/s/0646bc0aa66251909e74; accessed on 1 March 2021).
